# A group randomized controlled trial integrating obesity prevention and control for postpartum adolescents in a home visiting program

**DOI:** 10.1186/s12966-015-0247-8

**Published:** 2015-06-26

**Authors:** Debra L. Haire-Joshu, Cynthia D. Schwarz, Sarah B. Peskoe, Elizabeth L. Budd, Ross C. Brownson, Corinne E. Joshu

**Affiliations:** Washington University in St. Louis, The Brown School of Social Work and Public Health, and The School of Medicine, 1 Brookings Dr, St. Louis, MO 63130 USA; Department of Epidemiology, Johns Hopkins Bloomberg School of Public Health, Baltimore, MD 21218 USA; Harvard School of Public Health, 677 Huntington Avenue, Boston, MA 02115 USA

**Keywords:** Obesity prevention, Behavioral interventions, Adolescents

## Abstract

**Background:**

Adolescence represents a critical period for the development of overweight that tracks into adulthood. This risk is significantly heightened for adolescents that become pregnant, many of whom experience postpartum weight retention. The aim of this study was to evaluate Balance Adolescent Lifestyle Activities and Nutrition Choices for Energy (BALANCE), a multicomponent obesity prevention intervention targeting postpartum adolescents participating in a national home visiting child development-parent education program.

**Methods:**

A group randomized, nested cohort design was used with 1325 adolescents, 694 intervention and 490 control, (mean age = 17.8 years, 52 % underrepresented minorities) located across 30 states. Participatory methods were used to integrate lifestyle behavior change strategies within standard parent education practice. Content targeted replacement of high-risk obesogenic patterns (e.g. sweetened drink and high fat snack consumption, sedentary activity) with positive behaviors (e.g. water intake, fruit and vegetables, increased walking). Parent educators delivered BALANCE through home visits, school based classroom-group meetings, and website activities. Control adolescents received standard child development information. Phase I included baseline to posttest (12 months); Phase II included baseline to follow-up (24 months).

**Results:**

When compared to the control group, BALANCE adolescents who were ≥12 weeks postpartum were 89 % more likely (*p* = 0.02) to maintain a normal BMI or improve an overweight/obese BMI by 12 months; this change was not sustained at 24 months. When compared to the control group, BALANCE adolescents significantly improved fruit and vegetable intake (*p* = .03). In stratified analyses, water intake improved among younger BALANCE teens (*p* = .001) and overweight/obese BALANCE teens (*p* = .05) when compared to control counterparts. There were no significant differences between groups in sweetened drink and snack consumption or walking.

**Conclusion:**

Prevention of postpartum weight retention yields immediate health benefits for the adolescent mother and may prevent the early development or progression of maternal obesity, which contributes to the intergenerational transmission of obesity to her offspring. Implementing BALANCE through a national home visiting organization may hold promise for promoting positive lifestyle behaviors associated with interruption of the progression of maternal obesity.

**Trial registration:**

ClinicalTrails.gov NCT01617486.

## Introduction

More than one third of children and adolescents in the United States are overweight or obese [1]. Approximately 500,000 adolescents become pregnant each year enhancing their risk of developing obesity due to excessive gestational weight gain in this population [2, 3]. Studies have reported on average 27 % of adolescent mothers (≤19 years) gained more than 40 pounds during pregnancy, in contrast to 18 % of their adult counterparts; up to 20 % of adolescent mothers will retain or increase their pre-pregnancy weight by greater than 11 pounds [4]. Furthermore, weight retention associated with pregnancy is likely compounded with future pregnancies, resulting in a heightened risk for development of overweight, impaired glucose tolerance, type 2 diabetes, and other disease [3, 5]. Parental overweight supported by obesogenic behaviors, increases the risk for transmission of obesity from the adolescent mother to her child [6–8].

There is a dearth of empirical studies addressing postpartum interventions for overweight teens despite evidence that moderate weight reduction, even while breastfeeding, is safe [9, 10]. Postpartum women are often motivated to regain a prepregnant shape [11]. Major challenges to postpartum interventions are synchronizing intervention programs with the time constraints of new mothers, especially during the first few months postpartum [12].

Postpartum adolescents can benefit from energy balance interventions delivered in places where they spend time, such as home and school [13, 14]. Web-based, internet interventions have been shown to be an effective strategy for assuring dose and increasing physical activity and weight loss or maintenance in adults [15]. Web-based interventions are also a strategy for ensuring ongoing intervention dose, especially with the new demands of parenthood faced by this high-risk teen population [16]. Several studies have identified specific patterns that adversely influence weight in youth [17–19]. For example, daily consumption of sugared drinks has increased 300 % in 20 years and is associated with an increased obesity risk in young women [17, 18, 20, 21]. Increases in caloric density and number of snacks per day are a likely factor in the rise of youth overweight [22]. Expansion of food portion size in the marketplace over the past 20 years is another likely contributor to the obesity epidemic [23]. Adolescent females are also less likely to participate in vigorous, moderate, or team sports compared to males [24, 25].

Our research suggests parent education programs can be effective partners for improving energy balance behaviors. The High 5-Low Fat Program, a home based parent education intervention, significantly improved the dietary intake of African American parents [26]. The High 5 for Preschool Kids program demonstrated changes in parent dietary behavior were associated with improved child intake [14]. The purpose of this study was to test the impact of Balance Adolescent Lifestyle Activities and Nutrition Choices for Energy (BALANCE), a home + school + web-based parent education intervention designed to prevent or control obesity among postpartum adolescents. We hypothesized BALANCE adolescents would be more likely to maintain a healthy weight status or improve an unhealthy weight status when compared to control adolescents.

### Methods and procedures

BALANCE was developed in partnership with Parent As Teachers (PAT), a national parent education program delivered free of charge to over 200,000 families across all 50 states from the time of pregnancy until the youngest child in the home is 5 years of age. PAT services delivered by trained parent educators include child development screenings, home visits, on-site group activities, and newsletters. Parent educators typically reside in the communities they serve, are parents, and received a minimum of 30 h of parent education child development training before delivering PAT services to families. Parent educators also receive an additional two days of training to work with teen parents participating in the PAT teen program. This training includes learning to implement a specialized curriculum that supports teen development with emphasis on self-esteem, problem solving, goal setting, and decision-making. Services were generally offered in classes incorporated into the school day in addition to routine home visits. The ongoing contact and mentoring relationships between the parent educator and the teen parent has succeeded in keeping teen parents from dropping out of school, and has reduced the number of repeat pregnancies to below the national average [27, 28]. PAT teen programs served over 16,000 teen parents across the country in 2014 [29].

Consistent with our prior work, a community based participatory approach and extensive formative research guided the development of content and structure of BALANCE [14, 26, 30]. Methods to identify core content included developmental meetings with PAT staff, structured interviews, and pilot testing with teen parents. This approach, in collaborative partnership with PAT leaders and educators, assured the content were consistent with the mission, format, and procedures of the national organization and appropriate for teens. BALANCE used a combination of theoretical models to guide development including social cognitive theory and an ecological framework [31]. Intervention strategies targeted the intrapersonal environment of the teen (e.g., knowledge of high-risk patterns, self-assessment), interpersonal interactions among teen parents (e.g., group problem solving activities, teens as parent models of health behavior to babies), and the physical environment (e.g. improving school, home). Participatory methods were used to focus the intervention on replacing select high-risk patterns for teens (e.g. sweetened drink consumption, snack consumption, sedentary activity) with positive behaviors (e.g. water intake, fruit and vegetable snacks, increased walking) and ensure relevant content, materials, and survey measures. Consistent with the structure of the PAT Teen Program, BALANCE was comprised of three components to be delivered during the academic school year: home visits, school based classroom-group meetings, and internet activities.

#### Home visits

Parent educators were provided materials to conduct up to five 60-min BALANCE home visits focusing on a different behavior. At the first meeting with the teen parent the high-risk patterns were discussed to allow the parent educator to individualize visits relevant to teen’s behaviors and preferences. Subsequent home visits offered additional content while reinforcing prior information. Home visits meet required PAT organization elements: (ii) rapport building (e.g. concerns about teen’s health); (iii) self-assessment (e.g. individualized goal setting); (iv) observation of the parent, child, and/or home environment (e.g. assessment of access/availability to FV in home) (v) introduction of content (e.g. lifestyle change, skill building); (vi) parent–child activity to reinforce primary topic (e.g. parental modeling of positive eating patterns); (vii) reinforcement and follow-up (e.g. weekly feedback on goals, incentives). Consistent with the philosophy of the PAT program, each visit provided examples of parent–child activities designed around healthy nutrition and activity which the parent could use to promote the child's language and cognitive ability, and fine and gross motor skill development.

#### School based classroom-group meetings

The parent educator was provided materials to conduct up to five 60 min BALANCE classroom sessions focused on one behavior for teen moms as part of the school-based PAT program. The classroom lesson plans focused on (a) improving overall knowledge of high-risk patterns, (b) problem solving and goal setting scenarios, (c) providing hands on opportunities (i.e., taste testing, games, walking routes in school) to practice new behaviors, (d) and promoting social support for change.

#### BALANCE website

The teen was able to engage in a variety of ‘virtual’ interactive lessons delivered via the BALANCE web-based medium. Features of the website focused on (a) setting specific goals and feedback on progress in meeting those goals, (b) blogging with other BALANCE teens, (c) accessing activity, beverage and snack intake through a calorie calculator that allowed the teens to visually track progress, and (d) reinforcing knowledge through resources and educational tips. While there was no recommendation for website usage, parent educators encouraged teens to use the website during every visit. All handouts included the website information.

#### Parent educator training

Parent educators with the PAT teen programs were approached to complete the BALANCE training. The educators were located across 30 states; therefore a four hour training adapted from our prior work [13, 14] was developed with PAT trainers and conducted via videoconferencing technology. The training included: (a) background and rationale of BALANCE, (b) the relationship between diet and activity patterns and the health of the teen, (c) the application of social learning constructs to eating-activity changes, (d) the use of web-based activities to promote additional health behavior changes, and (e) review of curriculum lesson plans. Consistent with PAT philosophy and approach, the parent educator was responsible for, and trained, to assess and meet the priority needs of the teen mother during each contact. Therefore BALANCE was designed for flexible delivery to accommodate changing circumstances of teen mothers. Parent educators (*N* = 419, 12.4 % African American; mean age 38 ± 13) were evaluated based on 1) cognitive eating/activity pattern knowledge along with understanding and problem solving skills related to BALANCE and 2) satisfaction with the training. Those who successfully completed the training agreed or strongly agreed that video conferencing technology was effective as a means of training (78 %), and indicated that they would participate in another video conferencing training (97 %).

#### Sample recruitment

**Fig. 1:**
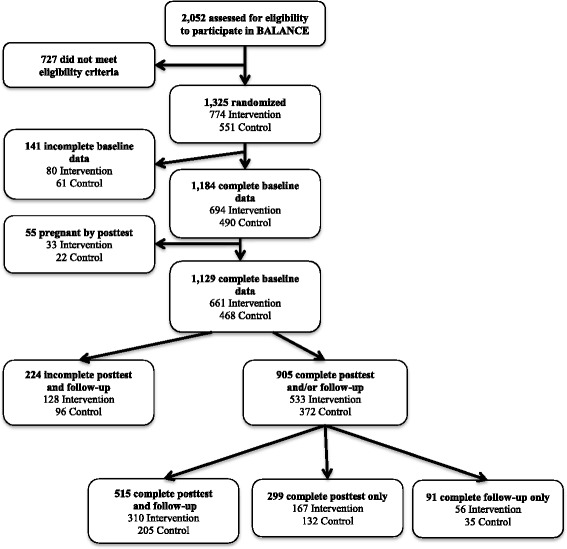
Consolidated standards of reporting diagram

BALANCE used a group randomized, nested cohort design using communities as the unit of allocation while the analysis is conducted at the level of the individual, controlling for the effect of secular trends on behavioral outcomes [32, 33]. Sample size calculations for 90 % statistical power were based on methods focused on nested cohort designs [34]. PAT affiliated adolescent programs across 30 states were divided into 3 strata based on number of adolescent parents expected in the state followed by random assignment. Other stratification criteria were not used since the demographics are similar for teen parents. Adolescents were eligible to participate if they were enrolled in the PAT Teen Program, were less than one year postpartum, and were not pregnant or planning to become pregnant. Eligibility and willingness to participate were assessed at the sites by the parent educator. Study staff followed up with interested adolescents to formally recruit and obtain consent. Across these sites, 1325 adolescents mothers were eligible to participate (774 randomized to intervention, 551 randomized to control) (Fig. 1). However, 141 of the 1325 participants randomized did not complete the baseline assessment, and 55 were pregnant at posttest. Of the 1129 participants that completed the baseline assessment and did not become pregnant, 905 participants completed a post and/or follow-up assessment. Among BALANCE participants with baseline assessments, those lost to follow-up were more likely to report their race/ethnicity as “Other” when compared with those who completed a post and/or follow-up assessments; there were no other significant differences in baseline characteristics. Among control participants with baseline assessments, there were no significant differences between those with and without post and/or follow-up assessments. The Institutional Review Board of Saint Louis University and Washington University in St. Louis approved this study. Informed consent was obtained from all participants in this study.

Data were collected across two phases: Phase I included baseline to posttest (12 months); Phase II included baseline to follow-up (24 months). Adolescent mothers completed the survey online; however 26 % did not have easy computer access and preferred to complete a paper version of the survey. There was no significant difference on the main outcome variable between online versus paper survey completers. Participants received a $15 gift card for completing the baseline survey and $30 gift card for completing the post survey. Retention approaches to ensure accurate contact information occurred via monthly phone call, email, or postcard contact. Staff first called participants and if unable to reach by phone, followed up via email and mailed postcards. Monthly attempts at contact continued until the end of the study period.

#### Program fidelity

Lesson content checklists were completed by the parent educator and used to monitor the delivery of BALANCE. Checklists provided a record on amount of parent educator contact with the adolescent, objectives covered, and information regarding materials and web-use. Parent educators rated each session on a scale of 1–5 (strongly disagree-strongly agree) regarding content relevance, parent participation, feedback, or progress toward meeting individual goals. Observation of random visits was conducted by research staff to assure reliability of self-assessment and intervention fidelity.

#### Outcome measures

Participants completed questionnaire and anthropometric assessments in designated private areas within school settings. Participants self-reported demographic and other characteristics including age, race/ethnicity, education, pre-pregnancy weight, postpartum status, and breastfeeding status. Trained staff measured height and weight in accordance with the National Health and Nutrition Examination Survey (NHANES) study procedures at baseline, post-test, and follow-up [35].

Weight and height data were used to calculate age-appropriate body mass index (BMI) at baseline, posttest, and follow-up. For participants up to 20 years of age, the percentile was calculated for BMI-for-age using the Centers for Disease Control and Prevention growth charts and algorithm [36]. Participants were classified as normal (<85th percentile), overweight (85th-94th percentile), or obese (≥95th percentile). For participants 20 years of age, BMI was calculated using height and weight data, and classified participants as normal (<25 kg/m2), overweight (25–29.9 kg/m2), or obese (≥30 kg/m2). To determine weight change, baseline weight was subtracted from weight at posttest and at follow-up. Weight change was not used as the primary outcome because the study population included (1) early postpartum participants who may be naturally losing weight, (2) younger participants who may be gaining weight as part of the growth process, and (3) normal weight participants not targeted for weight loss. Therefore the success of the intervention was conservatively evaluated based on the maintenance of a healthy weight status, or the improvement of an unhealthy weight status, using age-specific cut-points. Specifically at posttest and at follow-up, we categorized participants who were: (1) normal BMI for age at baseline as successful if they maintained normal BMI for age, and as unsuccessful if they became overweight or obese; (2) overweight for age at baseline as successful if they were normal BMI for age, and as unsuccessful if they were overweight or became obese; (3) obese for age at baseline as successful if they were normal BMI or overweight for age, and as unsuccessful if they remained obese.

Specific dietary behaviors were assessed at baseline and post-test only using the Snack and Beverage Food Frequency Questionnaire (SBFFQ), which was developed by selecting specific high calorie snack and beverage patterns of adolescents based on NHANES data [37]. An expert committee, including four registered dietitians, developed the SBFFQ for BALANCE following the format used from our previous work [14, 26] and the Diet History Questionnaire [38]. A validation study and pilot testing were completed with 60 teens. The SBFFQ examined adolescent intake of 31 items during the past seven days by asking how many days, how many times per day, and how much of the snack item the adolescent consumed. Intake was converted into the total kilocalories consumed for each food item and summed to obtain the total kilocalories of beverages and snacks. The test–retest were conducted within two weeks and reliability ranged from moderate to substantial [39] with the following ICCs: water (.71), sweetened beverages (.68), salty snacks (.43), meal type snacks (.64), fruits and vegetables (.46), and total kilocalories (.63). The difference was calculated in weekly consumption of food items between baseline and posttest. Participants were then categorized as to whether they increased water and fruit and vegetable consumption (any positive change in ounces or kilocalories consumed), and by whether they decreased intake of snack foods and sweetened beverages (any negative change in ounces or kilocalories consumed).

Physical activity was assessed at baseline and post-test only using a modified 3-Day Self-Administered Physical Activity Questionnaire [40]. Participants were asked to report on the type of activity performed on a weekend day and two weekdays, the length of time performing the activity, and the intensity at which they performed the activity. Test-retest were conducted within two weeks and reliability yielded ICCs of .29 for total activity and .26 for walking activity. The difference in activity was calculated between baseline and posttest. Participants were then categorized by whether they increased (any positive change in minutes) walking.

#### Statistical analyses

For the 1184 BALANCE and control participants with complete baseline data (Fig. 1), means and proportions were calculated for demographic and other factors by intervention status. Proportions were compared using chi-square tests and means using independent t-tests. Two approaches were used to evaluate study outcomes. First, the 1184 BALANCE and control participants with complete baseline data were compared using an intent-to-treat approach with multiple imputation for missing information. Because there was no statistical difference in baseline characteristics between participants with complete and incomplete post-test/follow-up assessments, we assumed missing at random [41]. Imputation was based on the estimated joint distributions of the randomized intervention group, race/ethnicity, time since baseline (for weight at posttest and weight at follow-up only), and the baseline values for age, weight, height, days postpartum, and the outcome of interest; and aggregated over 10 imputed sets. Next, the 905 BALANCE and control participants with complete baseline data and complete post and/or follow-up assessments were evaluated using a per protocol approach. More specifically, 814 participants (477 BALANCE, 337 control) were included in post-test analyses, and 606 participants (366 BALANCE, 240 control) were included in the follow-up analysis. For the intent to treat and per protocol approaches, logistic regression was used to estimate the odds ratio of (1) increased walking, (2) increased water consumption, (3) increased fruit and vegetable consumption, (4) decreased high calorie snack food consumption, (5) decreased sweetened beverage consumption, and (6) weight success by post-test comparing those in BALANCE to those in the control group. For the intent to treat and per protocol approaches, logistic regression was also used to estimate the odds ratio of, the odds ratio of weight success at follow-up comparing those in BALANCE to those in the control group. Two logistic models were compared using the likelihood ratio test: one accounting for PAT state, the unit of randomization, and one without PAT state; PAT state did not significantly improve the model, and was thus not included in the final analyses. All primary analyses were adjusted for age category (≤17 years, >17 years), race (non-Hispanic white, non-Hispanic black, Hispanic, other) baseline BMI status (normal weight, overweight or obese), and postpartum status (≤12 weeks, >12 weeks). In the primary analyses, participation in WIC and breastfeeding status did not appear to confounders and thus were not included in the final models. To explore whether participant factors modified the success of the intervention, primary analyses was stratified by participant age (≤17 years, >17 years), baseline BMI status (normal weight, overweight or obese), and postpartum status (≤12 weeks, >12 weeks). Participants in the intervention group were further categorized by number of visits (≤4 visits, >4 visits). All analyses were conducted using SPSS Version 17, 2008 (Chicago, IL) or SAS version 9.2 (Cary, NC). All tests were two-sided and results were considered statistically significant if *p* < 0.05.

## Results

At baseline, BALANCE adolescents were on average 17.7 years old, while the majority were non-Hispanic white (see Table 1). Approximately 41 % of adolescent mothers were less than 12 weeks postpartum at baseline. Adolescents in the control group were slightly older than BALANCE participants. There were no other significant differences between groups at baseline. BALANCE adolescents received a mean of 3.8 ± 2.7 home contacts (home visits mean: 2.5 ± 1.9; classroom visits mean: 1.3 ± 1.6) and visited the BALANCE website a mean of 4.2 ± 5.4 times.

**Table 1:** Demographic and baseline characteristics of the 1184 BALANCE and control participants with complete baseline assessments

	Intervention	Control	p-value
	*N* = 694	*N* = 490	
Age (years), mean (SD)	17.7 (1.3)	17.9 (1.3)	0.005
Race/Ethnicity, (%)			0.96
Non-Hispanic White	50.4	51.6	
Non-Hispanic Black	27.8	27.3	
White or Black Hispanic	19.4	18.4	
Other	2.4	2.7	
Participation in WIC, (%)	89.4	89.6	0.94
Breastfeeding, (%)	13.3	9.7	0.06
BMI category, %			0.19
Normal	58.7	53.5	
Overweight	23.2	25.3	
Obese	18.2	21.2	
Postpartum < 12 weeks, (%)	42.4	45.1	0.35
Total walking (minutes) per week, mean (SD)	114 (155)	128 (177)	0.18
Total water intake (grams) per day, mean (SD)	918.5 (969.6)	912.9 (938.4)	0.94
Total fruit and vegetable intake (kilocalories) per day, mean (SD)	62.3 (170)	57.1 (103.3)	0.57
Total snack intake (kilocalories) per day, mean (SD)	1,647.9 (1,077)	1,651.1 (1,041.6)	0.96
Total sweetened beverage intake (kilocalories) per day, mean (SD)	423.7 (439.4)	426.6 (394.1)	0.91

### 12-month posttest

BALANCE adolescents were not more likely than controls to report an increase in fruit and vegetable intake in intent to treat models overall or in stratified analyses. In per protocol analyses, BALANCE adolescents were 41 % more likely than controls to report an increase in fruit and vegetable consumption at posttest (*p* < 0.03, Table 2). In per protocol stratified analyses, BALANCE adolescents who were older (>17 years OR:1.58, *p* = 0.02) and earlier postpartum (≤12 weeks postpartum OR:1.70, *p* = 0.04) were significantly more likely to increase fruit and vegetable intake than their control counterparts. Improvements in fruit and vegetable intake were not impacted by number of intervention visits received (data not shown).

**Table 2:** Proportions by intervention status, and multivariable-adjusted odds ratios of behavioral and BMI success^a^

	Intent to Treat^b^			Per-Protocol^c^		
	% Success	OR^d^	95 % CI^d^	% Success	OR^d^	95 % CI^d^
Baseline to Posttest						
Increase Fruit & Vegetable Kilocalories						
Intervention	57.4	1.19	(0.89, 1.60)	49.9	1.41	(1.03, 1.92)
Control	52.9			41.8		
Increase Water Intake						
Intervention	52.8	1.10	(0.84, 1.44)	51.6	1.35	(0.98, 1.84)
Control	55.1			44.7		
Increase Walking						
Intervention	60.5	1.05	(0.82, 1.36)	55.9	1.14	(0.84, 1.55)
Control	59.1			53.0		
Decrease Total Snack Kilocalories						
Intervention	58.3	1.00	(0.77, 1.29)	60.3	0.97	(0.70, 1.33)
Control	58.3			61.1		
Decrease Sweetened Beverage Kilocalories						
Intervention	49.1	0.98	(0.75, 1.30)	53.1	0.99	(0.73, 1.36)
Control	49.8			53.1		
BMI Success^a^						
Intervention	65.0	1.27	(0.87, 1.86)	67.3	1.34	(0.90, 1.99)
Control	58.6			60.5		
Baseline to Follow-up						
BMI Success^a^						
Intervention	64.6	1.13	(0.78, 1.62)	66.3	1.20	(0.80, 1.82)
Control	61.2			64.6		

^a^BMI success defined as maintaining normal BMI at baseline, decreasing overweight BMI at baseline to normal BMI, or decreasing obese BMI at baseline to overweight or normal BMI

^b^Analysis includes 1184 participants with complete baseline data (694 BALANCE, 490 control). Multiple imputation conducted for missing data using ten imputed datasets

^c^Restricted to participants with valid (non-missing) outcome measures (Post-test: 477 BALANCE, 337 control; Follow-up: 366 BALANCE, 240 control)

^d^Odds ratio (OR) and 95 % confidence interval (95 % CI) adjusted for age, race, baseline BMI, and baseline postpartum status

BALANCE adolescents were not more likely than controls to report an increase in water intake in the intent to treat models overall or in stratified analyses. In per protocol analyses, BALANCE adolescents were 35 % more likely than controls to report an increase in water intake at posttest (*p* < 0.06, Table 2). In per protocol stratified analyses, BALANCE adolescents who were younger (≤17 years OR:2.93, *p* = 0.001) and those who are overweight or obese (OR:1.61 *p* = 0.05) were more likely to increase water intake than their control counterparts. Improvement of water intake was not impacted by number of intervention visits received (data not shown). When compared to controls, BALANCE adolescents were not significantly more likely to report an increase in walking, decrease in total snack kilocalories or sweetened beverage kilocalories (Table 2). These patterns were similar in intent to treat and per protocol analyses, overall and when stratified by baseline characteristics.

BALANCE adolescents were not more likely than controls to maintain a normal BMI or improve an overweight/obese BMI in intent to treat or per protocol models overall (Table 2). However, in stratified analyses, BALANCE adolescents who were ≥12 weeks postpartum were 61 % more likely in the intent to treat model (*p* = 0.06), and 89 % more likely in the per protocol model (*p* = 0.02), to maintain a normal BMI or improve an overweight/obese BMI (Table 3).

**Table 3:** Multivariable-adjusted odds ratios of BMI success^a^ at post-test by baseline characteristics

	Baseline to Posttest 12 months	
	Intent to Treat^b^	Per-Protocol^c^
	OR^d^ (95 % CI)	OR^d^ (95 % CI)
Normal Weight	1.31 (0.64, 2.67)	1.30 (0.58, 2.90)
Overweight or Obese	1.30 (0.78, 2.17)	1.40 (0.83, 2.30)
≤17 years	1.51 (0.71, 3.22)	1.33 (0.63, 2.81)
>17 years	1.22 (0.81, 1.85)	1.38 (0.86, 2.23)
≤12 weeks postpartum	0.90 (0.49, 1.64)	0.78 (0.41, 1.48)
>12 weeks postpartum	1.61 (0.98, 2.65)	1.89 (1.12, 3.20)
Low Intervention; ≤4 visits	1.21 (0.80, 1.85)	1.36 (0.86, 2.16)
High Intervention; >4 visits	1.44 (0.89, 2.34)	1.40 (0.84, 2.33)

^a^BMI success defined as maintaining normal BMI at baseline, decreasing overweight BMI at baseline to normal BMI, or decreasing obese BMI at baseline to overweight or normal BMI

^b^Analysis includes 1184 participants with complete baseline data (694 BALANCE, 490 control). Multiple imputation conducted for missing data using ten imputed datasets

^c^Restricted to participants with valid (non-missing) outcome measures (Post-test: 477 BALANCE, 337 control; Follow-up: 366 BALANCE, 240 control)

^d^Odds ratio (OR) and 95 % confidence interval (95 % CI) adjusted for age, race, baseline BMI, and baseline postpartum status

### 24-month follow-up assessment

BALANCE adolescents were not significantly more likely to maintain a normal BMI or improve an overweight/obese BMI by follow-up than controls though the odds ratios were in the positive direction (Tables 2).

## Discussion

In this high-risk, adolescent population, there was non-differential loss to follow-up. While intent to treat analyses with imputed information did not show significant differences, per protocol analyses identified several key findings that inform the literature on energy balance interventions conducted with postpartum teens mothers. First, BALANCE provides information to guide the initiation of interventions with postpartum adolescents for maximum success. At posttest, BALANCE teens who began the intervention at greater than 12 weeks postpartum were 89 % more likely (*p* = .02) to achieve or maintain a normal BMI than control teens. This improvement was apparently not sustained at 24-months which perhaps was due to the sizable loss to follow-up in this subset of very high-risk teens. Several studies report that the physical and psychosocial stressors of childbirth, including fatigue and role change, can significantly limit a woman’s ability for additional lifestyle changes during the postpartum period [20, 42, 43]. Lack of sleep, depression, and economic stressors may also influence a new mother’s priorities and further limit her ability to integrate lifestyle changes during the early postpartum period [43]. Obesity prevention interventions might be most effective if initiated with adolescent mothers after the 12 week postpartum phase, allowing some period of time to recover from the physical stressors of pregnancy and complete initial adjustments to parenthood.

Second, these results identify differences in the uptake of specific behavior changes, particularly among subsets of adolescent mothers. At posttest when compared to control adolescents, BALANCE was successful in improving fruit and vegetable intake (*p* = .03), showing particular effectiveness among older adolescents (*p* < .02) or those in the early postpartum period (*p* = .04); water intake improved among younger teens (*p* = .001) or those who were overweight or obese (*p* = .05). In contrast, BALANCE did not impact high-risk eating behaviors that required a reduction in intake (e.g. high fat snacks, sweet drinks), or required more of the teen’s time (e.g. walking). These findings suggest that changing lifestyle patterns that are continually reinforced through a variety of competing social and environmental systems, while always challenging, may be particularly so among new teen mothers [44]. Easy access to high fat snacks and sweetened beverages supported by media and marketing messages, may make it very difficult to limit or reduce high-risk eating patterns [23]. Lack of time, or other barriers such as safe places to walk, can also a challenge for women trying to add physical activity to daily routines making it a more difficult behavior to change [45]. The stressors of new parenthood further highlight the need to be attentive to determinants that may further complicate lifestyle behavior change. Consistent with the PAT approach, future interventions might prioritize behaviors the teen finds easiest to change, assuring adequate intervention and follow-up time to build on that success and confidence before introducing behaviors which appear more difficult.

It is also important to note that BALANCE ultimately improved quality of intake but was not able to document calorie reductions normally explained by some type of reduction in food or drink intake or increases in physical activity. The significant improvement in 12 month BMI suggests other dietary or activity changes were occurring which were not captured by our measures (e.g. reduction in portion size of meals due to increased water or vegetable intake; energy expended caring for the baby). The availability of robust measures appropriate for use with postpartum adolescents, and addressing the multiple social and other influences on obesity-related behaviors, is critical to understanding pathways through which modifiable factors can be addressed across environments targeted for obesity prevention [46].

Third, BALANCE shows the promise of designing research with a national partner that facilitates public health goals while benefiting the organization, addressing barriers to adoption and scaling-up the program for dissemination [47, 48]. Recent studies with postpartum women cite mixed impact on weight control due to limited participation [20, 49, 50]. Carter-Edwards et al. [51] reported that postpartum women want to participate in weight control interventions, but could not attend meetings and groups due to competing time commitments and responsibilities. BALANCE overcame this barrier by bringing energy balance strategies to the adolescent in their home and at school by partnering with a parent education program. However, the competing priorities of these very high needs adolescents were many and variable, which prevented or delayed the delivery of BALANCE by parent educators at scheduled times. As a result, BALANCE teens averaged 3.8 home contacts which generally reflects the level of participation reported by other community studies [13, 14]. It is unclear how many visits are needed to achieve optimal behavior change. However, our findings suggest immediate individual and parental needs, in addition to intervention structure, are major drivers of participation. This information can be used to better allocate resources to initiate and deliver BALANCE for maximum impact, yielding more appropriate allocation of staff and potential savings in organizational costs.

Strengths of this study include the group-randomized design that provides outcome data on a large group of postpartum adolescent girls. This was a national sample but only of adolescents enrolled in a parent education program, which limits generalizability of findings. Other limitations of this study include the use of self-report measures such as food frequency and physical activity questionnaires, that may yield error in outcome estimates or result in bias. This was a national versus locally based intervention limiting our ability, due to cost and access, to use more sensitive diet or physical activity assessment methods. However we took appropriate steps to assure the relevance and psychometric quality of our measures. Additional limitations included smaller sample size than estimated due to attrition in both groups and small numbers for sub-group comparisons. Because the loss to follow up was non-differential both in frequency and in baseline characteristics, we do not anticipate that this loss substantially biased our results.

## Conclusions

BALANCE adolescents who were greater than 12 weeks postpartum were more likely to maintain or improve an unhealthy weight status when compared to controls. Adolescent mothers who practice healthy lifestyle behaviors are more likely to maintain optimal weights associated with better outcomes in subsequent pregnancies, and to promote healthy eating and activity patterns to their offspring, interrupting the cycle of intergenerational obesity. Implementing BALANCE through a national home visiting organization holds promise for preventing the progression of maternal obesity.

## Abbreviations

BALANCE: Balance adolescent lifestyle activities and nutrition choices for energy
BMI: Body Mass Index
PAT: Parent as teachers
NHANES: National Health and Nutrition Examination Survey
SBFFQ: Snack and Beverage Food Frequency Questionnaire
